# Cognitively Demanding Object Negotiation While Walking and Texting

**DOI:** 10.1038/s41598-018-36230-5

**Published:** 2018-12-14

**Authors:** Preeti Chopra, Darla M. Castelli, Jonathan B. Dingwell

**Affiliations:** 1 University of Texas at Austin, Department of Kinesiology & Health Education, Austin, TX 78712 USA; 2 Present Address: Department of Kinesiology, Pennsylvania State University, University Park, PA 16803 USA

## Abstract

Cell phone related pedestrian injuries are increasing, but the underlying causes remain unclear. Here, we studied how cell phone use directly affected obstacle avoidance ability. Thirty healthy adults participated. Cognitive capacity was quantified using standard tests. Participants walked on a treadmill in a virtual reality environment with and without performing a texting-like cell phone task. Participants also navigated either ‘no’, ‘simple’ or ‘complex’ object negotiation tasks that directly manipulated the cognitive complexity of this object negotiation task. Cell phone use led to more collisions, delayed responses, and increased variability of responses when navigating objects. Mean object avoidance responses were further delayed for the cognitively more complex object negotiation task. Individuals’ baseline attentional capacity inversely predicted the number of object collisions when participants used the cell phone. Individuals with higher cognitive flexibility (i.e., better ability to switch between tasks) performed better on the cell phone task when they had to negotiate obstacles. Importantly, cognitive ability predicted performance only when both tasks (texting and negotiating objects) were being performed. Thus, using a cell phone while walking introduces a visual distraction that impairs healthy adults’ ability to respond to cognitively demanding object negotiation tasks in their environment.

## Introduction

The number of smart phone users in the US increased more than 3-fold from 2010 to 2016^1^. More than 25% of pedestrians used a handheld device in a busy street^2,3^. Similarly, 43.2% of pedestrians used a handheld device at an intersection on a University Campus^4^. These epidemiology studies make clear that many people use cell phones while walking^2–4^. Individuals who are distracted by texting on a cell phone while walking are 3.9 times more likely to exhibit at least one unsafe road crossing behavior (e.g. failing to look both ways, not following signal, etc.)^5^. Emergency room data for the US showed a dramatic increase in the number of pedestrian injuries due using cell phones while walking, increasing from 250 in 2005 to 1,500 in 2010^6^. These numbers do not include injuries treated outside emergency rooms. Thus, the actual number of such injuries is likely much higher. To mitigate these harmful effects of cell phone distraction among pedestrians, various authorities have passed laws to deter pedestrians from using cell phones while walking^7^. Despite the large number of injuries and such efforts, pedestrians often engage in using a handheld device^2–4^. While both road-related and behavioral factors contribute, few systematic studies have quantified underlying causes^5,8^. Therefore, it is critical to determine how cell phone use affects pedestrians’ ability to avoid collisions while walking.

Pedestrian cell phone use induces ‘inattentional blindness’^9,10^, that leads to failure to perceive unusual objects in the surrounding. Pedestrians using cell phones become less aware of nearby objects^9–12^, such as a clown riding a unicycle^9^ or money hung on a tree branch^10^. Cell phone use while walking also leads to a decrease in visual attention to the information about the path one is walking on^13^. Additionally, pedestrians who are using cell phones are less likely to follow safety measures^3^, which increases unsafe walking behavior. However, these observational studies were conducted in natural environments where variations in the number of obstacles (such as people, cars etc.) and other environmental factors could not be controlled and this might have affected responses^3,9,10,13^. Additionally, objects were placed near walking paths and not directly on the paths themselves^3,9,10,13^, so the possibility of actual collisions with these objects was not studied. Conversely, injuries occur due to collisions with objects that directly cross the walker’s path. Thus, it is critical to study distracted pedestrian behavior under controlled conditions where collisions with other objects are a real possibility.

Biomechanical studies conducted in controlled laboratory conditions showed that pedestrians alter their walking movements when using a cell phone^11,14–16^. Using a cell phone leads to slower walking speeds^17–20^, and decreased cadence^18,20,21^, stride lengths^18,22^ and step lengths^18,20^. These gait changes are consistent with using more “cautious” walking strategies while using a cell phone^11,15,16^, possibly to improve dynamic stability^14,16,23^. These effects may scale with either increasing cognitive difficulty of the cell phone task^24^ or increased physical difficulty of the walking task^25^. However, most gait changes can be attributed to walking slower and altering body posture to handle the phone^26^ and may possibly even disappear over time with adaptation to the task^27^.

Both vision^28,29^ and cognition^30^ play significant roles in regulating walking movements. Nevertheless, how visual and cognitive distractions introduced by cell phones and/or the environment affect walking has received little attention. Individuals with higher processing speed can cross a street more successfully^31^. Cognitive capacity such as attention and executive function (including cognitive flexibility, inhibition and working memory^32,33^) affect walking performance^34^. Importantly however, none of these biomechanical studies were designed to assess how people responded to *obstacles* while walking and thus were not designed to assess collision avoidance. Consequently, the extent to which the various components of cognitive capacity may affect (either separately or in combination) obstacle negotiation and texting performance remains largely unclear.

When *not* using cell phones, healthy pedestrians alter their walking strategies to avoid collisions with other pedestrians, even when not using cell phones^35–37^. Likewise, visual information is critical for either avoiding (unwanted) collisions or achieving successful interception tasks (like catching a ball, etc.)^38^. Thus, the inattentional blindness caused by using a cell phone^9–12^ should lead to increased collisions. Indeed, during a simulated pedestrian crossing, people were slower and more variable when avoiding two oncoming pedestrians than one^39^. People slowed down and reduced their stride length when texting and having to negotiate obstacles that could be stepped over^22,40^ or around^41^. In each of these studies, neither time nor speed were constrained, and healthy participants avoided or navigated the obstacles presented primarily by slowing down and adapting their gait accordingly. However, pedestrians often either may not have the option to slow down (e.g., when running to catch a bus) or may face obstacles that appear unexpectedly. To our knowledge, no study has yet examined pedestrians’ responses to obstacles that require pedestrians to change their walking path (e.g. car, other people, pole, pothole etc.) under realistic time or speed constraints.

Overall, there is a lack of research regarding how using a cell phone affects pedestrians’ ability to avoid collisions^5,8^ and existing findings are equivocal. First, there are few studies conducted under controlled conditions. Second, pedestrians’ responses to obstacles that can only be avoided by changing their walking path has not been studied. Third, the effect of the complexity of the obstacle avoidance task on performance is not known. Finally, little is known about the extent to which an individuals’ cognitive capacity affects their ability to negotiate objects while using cell phones. Avoiding obstacles while walking and texting is a complex process involving multiple interacting tasks (walking, texting, navigating one’s environment). To date, many of these components have been studied separately, but they have not been studied in combination in a controlled and systematic way. By doing so, we can better understand how these different sub-tasks interact with each other to either cause or avoid potentially injurious collisions.

Therefore, this study quantified how performing a texting-like task on a cell phone affected healthy persons’ ability to negotiate objects (avoid obstacles or intercept targets) in their path. We hypothesized that pedestrians would take more time and would fail more often to respond to oncoming objects when using a cell phone. Moreover, we quantified how an increase in the cognitive demand of the object negotiation task affected these performances. Conversely, the cell phone task performed was chosen specifically to require *minimal* cognitive demand, creating instead a primarily visual distraction. Additionally, we hypothesized that performance on both cell phone task and object negotiation task would decline due to an increase in the cognitive complexity of the object negotiation task. Lastly, we hypothesized that individuals with better baseline cognitive ability would perform better on both the cell phone task and the object negotiation task.

## Results

Our goal was to determine how the increasingly common use of cell phones during walking affects individuals’ responses to approaching obstacles. Human participants walked on a treadmill in a virtual reality environment (see Methods, Fig. 7) and navigated different combinations of randomly appearing objects while playing or not playing a texting-like game (see Methods) on a cell phone. During the object negotiation tasks, participants had to avoid obstacles (red balls) or hit targets (green balls) by shifting lanes laterally. During the “Simple” object negotiation task, only obstacles were presented. During the “Complex” object negotiation task, both obstacles and targets were presented. The Complex task was cognitively more challenging because participants had to make an additional decision (i.e., obstacle or target) based on the color of the object. We introduced a texting-like task (a game app called ‘Fish Farts’; see Methods) to provide a visual distraction that was cognitively minimally challenging. However, the visual and biomechanical demands during both object negotiation tasks were similar. All objects (obstacles and targets) were spheres of the same visual size. Both obstacles and targets were presented in the same locations relative to the treadmill, with the same relative timing, and differed only by color (red vs. green). Likewise, the biomechanical response participants were asked to perform was similar in both tasks: move to the other walking lane when appropriate.

Each participant performed 6 tasks: No Texting plus either No (NN), Simple (NS), or Complex (NC) object negotiation, and Texting plus either No (TN), Simple (TS), or Complex (TC) object negotiation. Percent Collision, Mean of Movement Time (Mean MT) and Standard Deviation of Movement Time (SD MT) were calculated for each object condition (NS, NC, TS and TC). Game Scores were calculated for each of the three conditions that involved the using a cell phone (TN, TS and TC). Baseline cognitive ability was measured in terms of Reaction Time (RT), Failure to Maintain Set (FMS), Perseverative Error and Perseverative Response. Baseline cognitive measures and participant demographics are shown in Table 1.

**Table 1:** Demographics and baseline cognitive measures of participants.

Characteristic:	Value:
Sex (M/F)	14 M/16 F
Age (years)	21.73 ± 3.56
Body Mass (kg)	66.09 ± 14.39
Body Height (m)	1.71 ± 0.10
BMI (kg/m^2^)	22.53 ± 4.19
Leg Length (m)	0.92 ± 0.06
Walking Speed (m/s)	1.20 ± 0.31
Intelligence Quotient (IQ)	101.2 ± 11.41
Reaction Time (msec)	336.03 ± 39.53
Failure to Maintain Set	1.17 ± 1.34
Perseverative Response (%)	32.47 ± 4.91
Perseverative Error (%)	11.88 ± 5.10

Thirty healthy participants age 18–29 years participated. Walking speeds were set based on leg length (see Methods). Intelligence Quotient was calculated from Kaufman Brief Intelligence Test (KBIT-2), Reaction time was calculated from PEBL Perceptual Vigilance Task (PPVT); Failure to Maintain Set, Perseverative Response and Perseverative Error were calculated from Berg’s Card Sorting Test (BCST).

### Game Score

**Figure 1:**
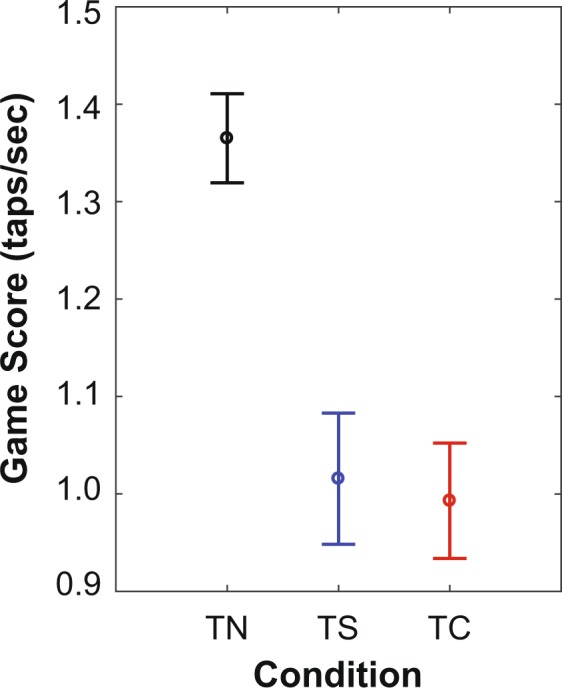
Cell Phone Task Performance During Different Negotiation Tasks. Mean Game Scores during Texting + No Negotiation (TN), Texting + Simple Negotiation (TS) and Texting + Complex Negotiation (TC). Error bars represent between-subject 95% confidence intervals. Game Scores during TN (Mean = 1.37, 95% CI = 1.32–1.41) were significantly greater (p < 0.001) than during TS (Mean = 1.02, 95% CI = 0.95–1.08) or TC (Mean = 0.99, 95%CI = 0.93–1.05). However, Game Scores during TS and TC were not different (p = 0.85).

Game Scores (Fig. 1) were calculated as total number of accurate finger taps per second and quantified performance on the cell phone task. Higher Game Scores corresponded to better performance. The cell phone task performance decreased significantly (p < 0.001) when participants had to negotiate objects (TS and TC) relative to when there were no objects (TN). Tukey post-hoc analysis demonstrated that Game Scores during TN were significantly higher than both TC (p = 0.00) and TS (p = 0.00), but were not different between TS and TC (p = 0.85) (Fig. 1).

### Percent Collision

**Figure 2:**
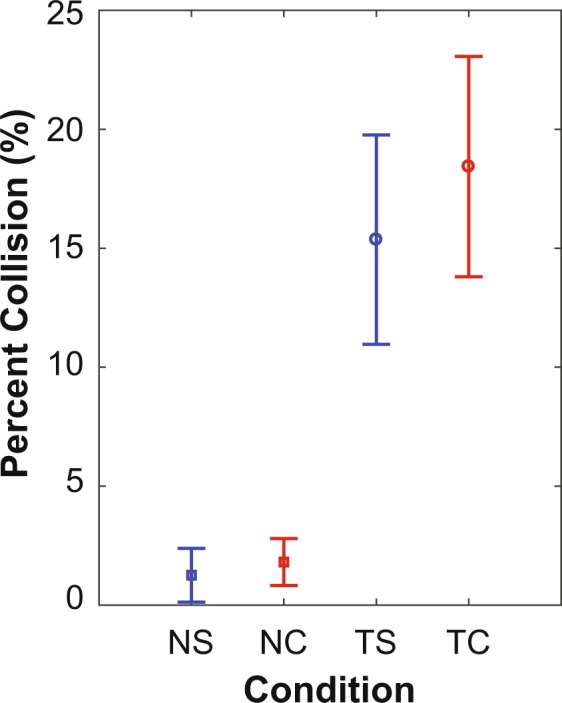
Object Negotiation Performance During Simple and Complex Negotiation Tasks. Mean Percent Collision during No Texting + Simple Negotiation (NS), No Texting + Complex Negotiation (NC) Texting + Simple Negotiation (TS) and Texting + Complex Negotiation (TC). Error bars represent between-subject 95% confidence intervals for each mean. Percent Collision for No Texting conditions, NS (Mean = 1.26, 95% CI = 0.08–2.44) and NC (Mean = 1.81, 95% CI = 0.78–2.84) was significantly lower (p < 0.001) than Percent Collision for Texting conditions, TS (Mean = 15.36, 95% CI = 10.77–19.95) and TC (Mean = 18.43, 95% CI = 13.60–23.27). Nevertheless, Percent Collision was unaffected by the complexity of the negotiation task (p = 0.28). There was no Texting × Negotiation interaction (p = 0.45).

Percent Collision (Fig. 2) quantified failure to respond correctly to objects. This was calculated as the number of failures as a percent of the total number of objects presented. Lower Percent Collision represents better performance on the object negotiation task. Percent Collision was significantly greater (p < 0.001) when texting, but did not differ between the two different object negotiation tasks (p = 0.28; Fig. 2). Additionally, there was no significant Texting × Negotiation interaction (p = 0.45) between cell phone use (texting vs. not) and the object negotiation task (simple vs. complex) (Fig. 2).

### Movement Time

**Figure 3:**
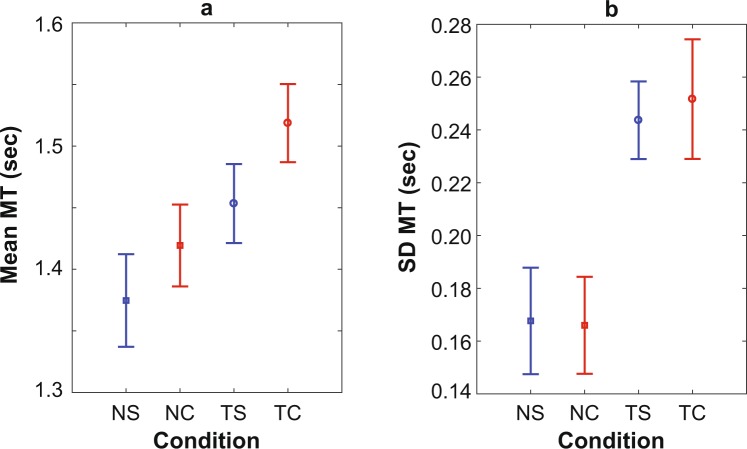
Mean and Standard Deviation of Movement Time. Movement Time (MT) was the time taken to negotiate an object. MT was calculated for No Texting + Simple Negotiation (NS), No Texting + Complex Negotiation (NC) Texting + Simple Negotiation (TS) and Texting + Complex Negotiation (TC). All error bars represent between-subject 95% confidence intervals for each mean. (**a**) Within-subject average of MT (Mean MT). Mean MT during TS (Mean = 1.45, 95% CI = 1.42–1.49) and TC (Mean = 1.52, 95% CI = 1.49–1.55) were higher (p < 0.001) than Mean MT during NS (Mean = 1.37, 95% CI = 1.34–1.41) and NC (Mean = 1.42, 95% CI = 1.39–1.45). Additionally, Mean MT was higher for Complex as compared to Simple Negotiation (p = 0.002). There was no significant Texting × Negotiation interaction effect (p = 0.43). (**b**) Within-subject standard deviation of MT (SD MT). SD MT was significantly higher (p < 0.001) during Texting, TS (Mean = 0.24, 95% CI = 0.23–0.26) and TC (Mean = 0.25, 95% CI = 0.23–0.27) as compared to No Texting, NS (Mean = 0.17, 95% CI = 0.15–0.19) and NC (Mean = 0.17, 95% CI = 0.15–0.18). SD MT was not affected by the complexity of the negotiation task (p = 0.75). Additionally, there was no Text × Negotiation interaction (p = 0.62). SD MT determines variability in MT. Consequently, MT was more variable when participants were Texting but did not alter by the complexity of the negotiation.

Movement Time (MT; Fig. 3) was the time taken by an individual to respond to each object in a given condition. Mean of Movement Time (Mean MT; Fig. 3a) was calculated as the average of MT across all objects of each condition. Time to negotiate objects increased significantly (p < 0.001) when playing the cell phone texting game. Movement Times were also significantly higher (p = 0.002) for Complex Negotiation as compared to Simple Negotiation of oncoming objects. These effects of texting and the object negotiation tasks were independent: there was no significant Texting × Negotiation interaction effect (p = 0.55; Fig. 3a).

Standard Deviation of Movement Time (SD MT; Fig. 3b) was calculated as the standard deviation of the MT exhibited by a participant, across all objects of each condition. SD MT was significantly higher during both conditions (TS and TC) that involved playing the cell phone texting game (Fig. 3b). However, it was not affected by the complexity of the object negotiation task (i.e. Simple vs. Complex) (p = 0.75). Additionally, there was no significant Texting × Negotiation interaction effect between cell phone use and complexity of the object negotiation task (p = 0.62; Fig. 3b).

### Correlations Between Baseline Cognitive Ability and Task Performance

As shown above, performance on the cell phone and the object negotiation tasks differed across different conditions. This indicated that the six experimental conditions were distinct from each other. Therefore, linear regression analyses of baseline cognitive ability scores with the performance on the cell phone and the negotiation tasks were conducted separately for each experimental condition.

#### Cognitive Ability and Object Negotiation

**Figure 4:**
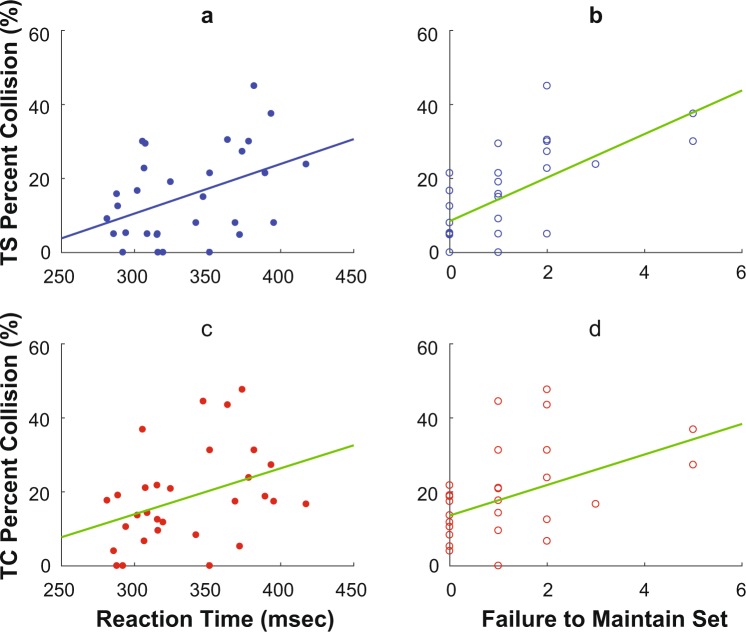
Cognitive Behavior Predicts Object Collisions While Using Cell Phone. Within each plot, each data point represents one individual participant. Straight lines indicate linear regression fits. Percent Collision during Texting + Simple Negotiation (TS) was positively predicted by both (**a**) Reaction Time (RT) during PEBL Perceptual Vigilance Task (PPVT) (r^2^_Adj_ = 0.16, p = 0.017) and (**b**) Failure to Maintain Set on Berg’s Card Sorting Test (BCST) (r^2^_Adj_ = 0.39, p < 0.0005). Percent Collision during Texting + Complex Negotiation (TC) was likewise positively predicted by both (**c**) Reaction Time (r^2^_Adj_ = 0.11, p = 0.039) and (**d**) Failure to Maintain Set (r^2^_Adj_ = 0.15, p < 0.019).

Individuals with slower reaction times (RT) and higher Failure to Maintain Set exhibited higher Percent Collision while both texting *and* negotiating objects (r^2^_Adj_ ≥ 0.11, p ≤ 0.04; Fig. 4). When using the cell phone, Reaction Time and Failure to Maintain Set both predicted Percent Collision during both Simple and Complex negotiation tasks (Fig. 4). Conversely, when *not* texting, RT trended towards being correlated to Percent Collision during NS (r^2^_Adj_ = 0.10, p = 0.053), but not during NC (r^2^_Adj_ = -0.02, p = 0.47) (not shown). Failure to Maintain Set was also not correlated to Percent Collision during NC (r^2^_Adj_ = –0.03, p = 0.72). Although Percent Collision during NS was significantly correlated to Failure to Maintain set (r^2^_Adj_ = 0.12, p = 0.03), this finding was considered spurious since Percent Collision during NS was zero for 25 out of 30 participants.

#### Cognitive Ability and Texting Performance

**Figure 5:**
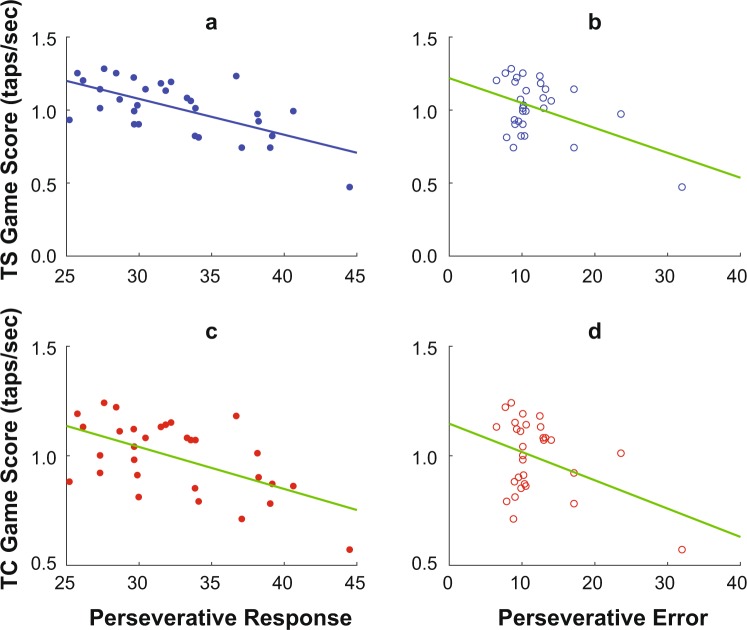
Cognitive Behavior Predicts Game Score While Negotiating Objects. Within each plot, each data point represent one individual participant. Straight lines indicate linear regression fits. Participants’ cell phone task Game Scores during Texting + Simple Negotiation (TS) were predicted by both (**a**) lower Perseverative Responses (r^2^_Adj_ = 0.39, p < 0.0005) and (**b**) lower Perseverative Errors (r^2^_Adj_ = 0.185, p = 0.010) and on Berg’s Card Sorting Test (BCST). Game Scores during Texting + Complex Negotiation (TC) were likewise strongly predicted by both (**c**) lower Perseverative Responses (r^2^_Adj_ = 0.30, p = 0.001) and (**d**) lower Perseverative Errors (r^2^_Adj_ = 0.13, p = 0.029) and on Berg’s Card Sorting Test (BCST).

Perseverative Response and Perseverative Error (see Methods) measured at the baseline predicted individual’s performance on the cell phone task while both texting *and* negotiating objects (r^2^_Adj_ ≥ 0.13, p ≤ 0.03; Fig. 5). Game Scores were higher for individuals with low Perseverative Error and low Perseverative Response. When *not* negotiating objects, Game Scores during TN trended towards being correlated to Perseverative Response (r^2^_Adj_ = 0.11, p = 0.07) or Perseverative Error (r^2^_Adj_ = 0.12, p = 0.06), but these did not reach statistical significance.

## Discussion

Use of cell phones has increased dramatically in recent years^1^ and many people regularly use cell phones while walking^2–4^. Using a cell phone can distract people from things going on around them^9–12^. This may contribute to the sharp increase in pedestrian accidents attributable to cell phone use^6^. However, no studies to date have systematically studied how performing a texting-like task on a cell phone alters both walking performance and the ability to negotiate interactions with objects in one’s environment. This study determined how performing a simple texting-like task on a cell phone would affect healthy participants’ ability to negotiate various obstacle interaction tasks in their environment and to determine the extent to which these peoples’ baseline cognitive ability predicted their performance.

The present study imposed *virtual* objects to either avoid (obstacles) or intercept (targets). There was never any real risk of an actual collision with a real physical object. Participants could have been well aware of this and responded to these virtual objects quite differently than they might otherwise respond to real objects encountered in real circumstances (which might impose potentially much more dangerous consequences). However, the VR environment used here was highly immersive^42^. All participants did respond to the objects presented and all responded exactly as one would expect them to had these been real objects: i.e., they clearly tried to avoid virtual “collisions”. At no point did any participant “ignore” the oncoming virtual objects, even though they easily could have with no actual risk of injury. Our data fully substantiate this observation. Participants exhibited highly significant differences in Mean MT across all conditions (Fig. 3a). However, while Mean MT was significantly longer for both NC vs. NS and for TC vs. TS (Fig. 3a), there were no corresponding differences in% Collision between these conditions (Fig. 2). Thus, during both NC and TC, participants achieved the same net success rate (% Collision) as for TS and NS, albeit with slower movement times. This means that during both NS and TS, participants could have been equally successful at avoiding collisions without responding as quickly as they did. That they did respond more quickly than they needed to indicates that they were highly responsive to the *perceived* threat of colliding with the virtual objects presented to them. This strongly suggests participants most likely would have responded very similarly in situations involving actual physical objects that imposed a threat of real collisions.

Texting speed decreases when performing an additional task that involves vision^11^. Similarly, we found a significant decrease in Game Scores due to the simultaneous object negotiation tasks (Fig. 1). Participants had to shift vision away from the phone to see objects coming towards them during both Simple and Complex Negotiation. This shift of visual attention led to a decrease in Game Scores during these conditions. The cognitive complexity of the object negotiation task did not affect Game Scores. This could be due to the minimally cognitively challenging nature of the cell phone task.

Texting while walking led to a decrease in the ability to avoid an obstacle (i.e., more collisions; Fig. 2) and in the variability in the time taken to respond to approaching objects (Fig. 3b). Cell phone use leads to a delay in detecting visual signals by pedestrians^19^, which might have contributed to these increased movement times. Additionally, ability to avoid an obstacle decreases due to an additional visual task^40,43^. Thus, Percent Collision and Movement Time, which each quantified the inability to navigate, both increased most likely due to the visual distraction introduced by the texting task (Fig. 2). Unlike texting trials, participants were not required to look at the phone during non-texting trials. This allowed participants to maintain continuous visual attention on the environment during non-texting trials. Thus, participants could perceive objects as soon as they appeared on the screen during non-texting trials, leading to both faster overall movement times (Fig. 3a) and less variability in their movement responses (Fig. 3b).

Individuals had to shift visual attention between the cell phone and the environment (road) to perform the texting and the negotiation task simultaneously. The obstacles were presented at random time intervals. Thus, shifting of visual attention likely led to greater variation in the time to perceive an obstacle, which in turn likely increased the overall variability of movement time (Fig. 3b). Overall, using a cell phone while walking reduces the ability of a pedestrian to avoid an obstacle and increases the variability in response. Most previous studies have assumed it is some cognitive demand of texting on a cell phone that leads to increased risk of accidents etc^11,15,16,24^. Our findings, however, suggest the opposite: that it is instead a combination of simply looking at the phone (i.e., “inattentional blindness”^9,10^) and the complexity of the decisions that need to be made with respect to events going on in the environment (i.e., obstacle avoidance) that increase the risk for collision and potential injury. The cell phone task used in this study (which intentionally imposed *minimal* cognitive demand) impaired performance (Figs 2 and 3b) by requiring continuous switching of visual attention between the phone and the environment^14,40^. Conversely, it was the cognitive load not of the cell phone task, but of the object negotiation task that led to slower movement times (Fig. 3a), independent of whether participants were texting or not. Together, these results clearly demonstrate that the specific demands (physical and cognitive) of both tasks are critical in determining peoples’ overall task performance and success rates.

Ability to avoid an obstacle decreased due to an increase in the cognitive complexity of a secondary task^43^, but did not reach significance in that study^43^. Similarly, performing more complex walking tasks while texting degraded both walking and texting performance^25^ and people walked slower and with more variability when avoiding 2 vs. 1 other pedestrians while looking at (but not using) a cell phone^39^. In our study, the cognitive complexity of the negotiation task did not affect Percent Collision (Fig. 2), but did lead to increased mean Movement Times, independent of whether people were texting (Fig. 3a). Conversely, variability of Movement Time was not affected by the complexity of the object negotiation task (Fig. 3b). Most likely, it was the random switching of visual attention that led to greater variability in when objects were perceived, which then contributed to this increased SD MT (Fig. 3b). However, once perceived, participants had to decide upon an action (move or not move) and execute that action. As the required movement (move to the other lane) was the same (on average) for both object conditions (Simple vs. Complex), it was most likely the increased time required to make that decision in the Complex object negotiation conditions that led to the increased mean MT (Fig. 3a). Our findings thus extend prior studies^25,39^ to provide direct evidence that the task one has to negotiate in their environment is at least as important as the task being performed on the cell phone.

Individuals with better cognitive capacity performed better on both the cell phone and negotiation tasks while performing both tasks concurrently (Figs 4 and 5). Those with lower Reaction Time (RT) (i.e. faster processing speed) during PPVT had lower Percent Collision when using the cell phone (Fig. 4a,c). This is similar to previous findings that people with faster processing speed are more successful at crossing streets^31^. During texting while negotiating, people with higher Failure to Maintain Set during BCST had higher Percent Collision (Fig. 4b,d). Thus, the task of avoiding obstacles while using a cell phone may have required individuals to learn a pattern (e.g. to shift visual attention at a specific phase of the gait cycle) and follow that learned pattern to successfully respond to objects while using a cell phone. Individuals with higher Perseverative Error and Perseverative Responses are considered to have lower cognitive flexibility^44–46^. While texting and negotiating simultaneously, individuals with higher cognitive flexibility were better at the cell phone task (Fig. 5).

There is some possibility these correlations were spurious (i.e., they appeared to be “statistically significant” by random chance). Here, we obtained 7 total cognitive variables from our baseline tests and we computed a total of 7 × 15 = 105 correlations. However, prior to conducting these correlation analyses, we tested these for multi-collinearity and found it to be minimal: i.e., each of the 7 cognitive variables measured something different. Likewise, we correlated each of these predictors against 4 outcome variables (Game Score, % Collision, Mean MT, and SD MT), which were again largely independent of each other. These outcome variables were then obtained from 5 functionally different and also largely independent test conditions (TN, TS, TC, NS, NC). It is also notable that of the 105 total correlations computed, all 8 that were found to be statistically significant at p < 0.05 (Figs 4 and 5) occurred only for those conditions where participants were both texting *and* simultaneously negotiating objects in their path. Thus, these factors together substantially mitigate concerns about multiple comparisons and Type I error in these comparisons.

Correlations of Percent Collision with RT and Failure to Maintain Set were significant when negotiation tasks were performed simultaneously with the texting-like task. No correlations were significant when participants negotiated objects but were not texting, or when they were texting but not negotiating objects. Thus, cognitive capacity was more relevant when “triple-tasking” (both texting and negotiating objects while also walking) than when having to either just text or just negotiate objects while walking. Additionally, the negotiation task by itself may not have been cognitively challenging enough to elicit significant correlations. Moreover, correlations could be insignificant due to a ceiling effect under No Texting conditions. Out of 30 participants, 25 had no collisions during NS and 20 had no collisions during NC. This ceiling effect could be reduced by decreasing the amount of time available to respond, as ability to avoid an obstacle increases when time available to respond is high^43^. When participants were texting without negotiating, their performance was not correlated to cognitive flexibility. This is likely because cognitive flexibility is less relevant when individuals do not need to switch between tasks.

One participant tested at a Perseverative Error equal to 32.03 and appeared to be a possible outlier from the rest of the group. The correlations of Game Score during TS and TC with Perseverative Error each became non-significant after removing this participant. However, we did not remove this participant from the analyses because the observed Perseverative Error was close to 2 standard deviations of usually observed Perseverative Error, which typically has a Mean ± SD of 15.5 ± 7.8^46^.

Participants in this study were healthy individuals age 18 to 29 years, which is considered to be within the range of peak cognitive ability for most cognitive functions^47^. Thus, variability in cognitive capacity of individuals might have been lower as compared to individuals across different stages of the life span and/or different levels of cognitive impairment. Low variability in the cognitive capacity may have affected the correlation analyses of cell phone and object negotiation task performance with cognitive capacity. The present study was not designed to determine if similar effects of cell phone use might be seen in younger children, older adults and/or in persons with specific cognitive, visual and/or biomechanical impairments. Thus, studying the behavior of people with different cognitive abilities across different age ranges would help better elucidate the relationship between cognitive ability and obstacle avoidance performance.

Overall, we found that performing even a simple cell phone task while walking increased the likelihood of being hit by an obstacle. These effects were caused primarily by the visual distraction of having to look at the cell phone and not by the cognitive demand of the task being performed on the phone (which was purposefully kept minimal here). Moreover, we found that the cognitive complexity of the object negotiation task presented in the environment was equally and independently important in determining how quickly people could respond to object appearing in their path as using a cell phone. Additionally, individuals with better cognitive capacity were better able to perform both cell phone and negotiation task under multi-tasking conditions.

## Methods

### Participants

All methods and procedures performed were carried out in accordance with the relevant guidelines and regulations and were approved by the University of Texas at Austin Institutional Review Board. Prior to participating, all participants reviewed and signed a written informed consent form approved by the University of Texas IRB. Thirty young healthy adults, age 18–29 years, participated (Table 1). All participants were screened to ensure that they had no history of orthopedic, visual or neurological impairments or medications that would have affected their ability to perform the required walking and texting tasks.

### Baseline Cognitive Testing

The battery of cognitive tests consisted of the Kaufman Brief Intelligence Test – 2 (KBIT-2), PEBL Perceptual Vigilance Task (PPVT) and Berg’s Card Sorting Test (BCST). KBIT-2 was administered according to the standard protocol^48^. PPVT and BCST were administered on a laptop using Psychology Experiment Building Language (PEBL, Version 0.14) software^45^.

KBIT-2 measured Intelligence Quotient (IQ)^48^. The test consists of three sections: verbal knowledge, matrices, and riddles. Each section had multiple questions that required participants to respond in a word or select one of the multiple pictures presented to them. The verbal knowledge and riddles section measured verbal intelligence. The matrices portion of the test measured non-verbal intelligence.

**Figure 6:**
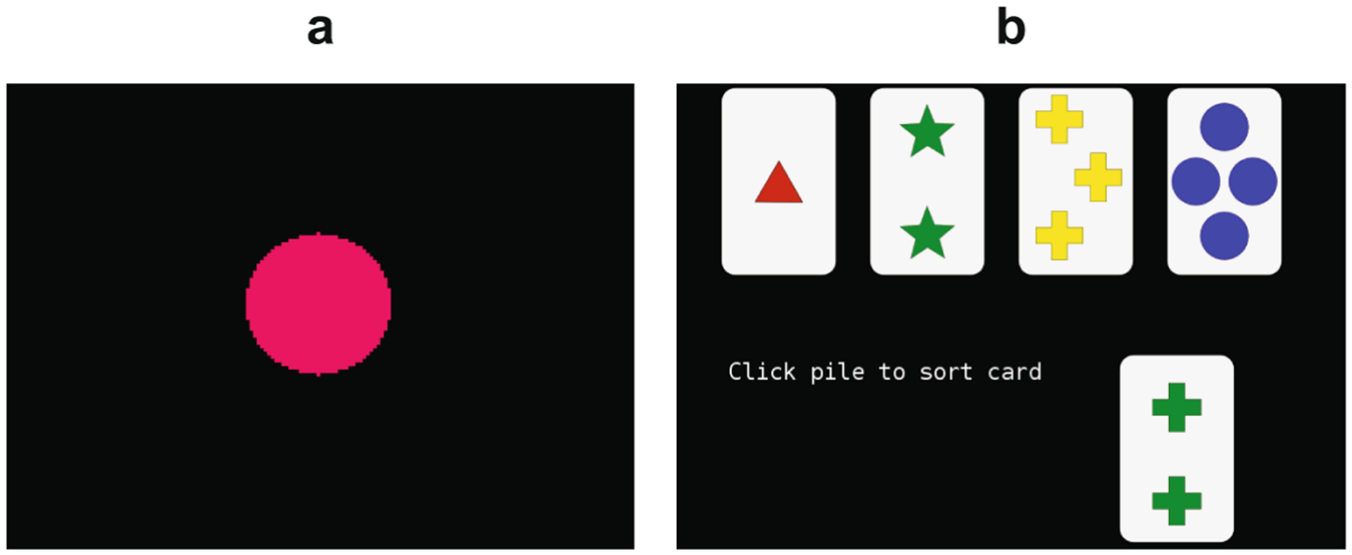
Cognitive Tests. (**a**) PEBL Perceptual Vigilance Task (PPVT) consisted of a single stimulus that appeared at randomly-varying time intervals. Participants responded to the stimulus as quickly as they could and Reaction Time (RT) was calculated. (**b**) Berg’s Card Sorting Test (BCST) consisted of a card on the lower row that had to be matched to one of the four cards in the top row, based on the rules (explained in the Method section). Perseverative Error, Perseverative Response, and Failure to Maintain Set were calculated based on the performance.

PPVT is a simple reaction time task used to measure attention^49^. The test consisted of 6 practice trials followed by 25 testing trials. At the beginning of each trial, a fixation cross ‘ + ’ (ready signal) was presented for 100 msec. After that, a stimulus (red circle; Fig. 6a) was presented at a random time interval. Participants responded to each stimulus by pressing the space bar as quickly as they could. The time lag between each stimulus and its response was recorded as a reaction time and was displayed after each response.

BCST is a PEBL version of the Wisconsin Card Sorting Test (WCST)^45^ that measures different components of executive function. The test was validated by Fox, *et al*.^46^. During the test, participants were presented with one card at a time. They had to use a mouse to match the card to one of the four cards that were different from each other in terms of color, shape, and number (Fig. 6b). The card had to be matched based on one of the three rules: i.e. same color, same shape, or same number. For each trial, only one rule was correct. After each response, participants received visual feedback indicating if their match was correct or not. To match correctly, participants had to figure out the correct rule. Participants first completed 10–12 practice trials where the rule changed after every 4 correct responses. After this acclimation, participants performed the full test that included approximately 120 trials where the rule changed after every 10 correct responses.

### Experimental Protocol

**Figure 7:**
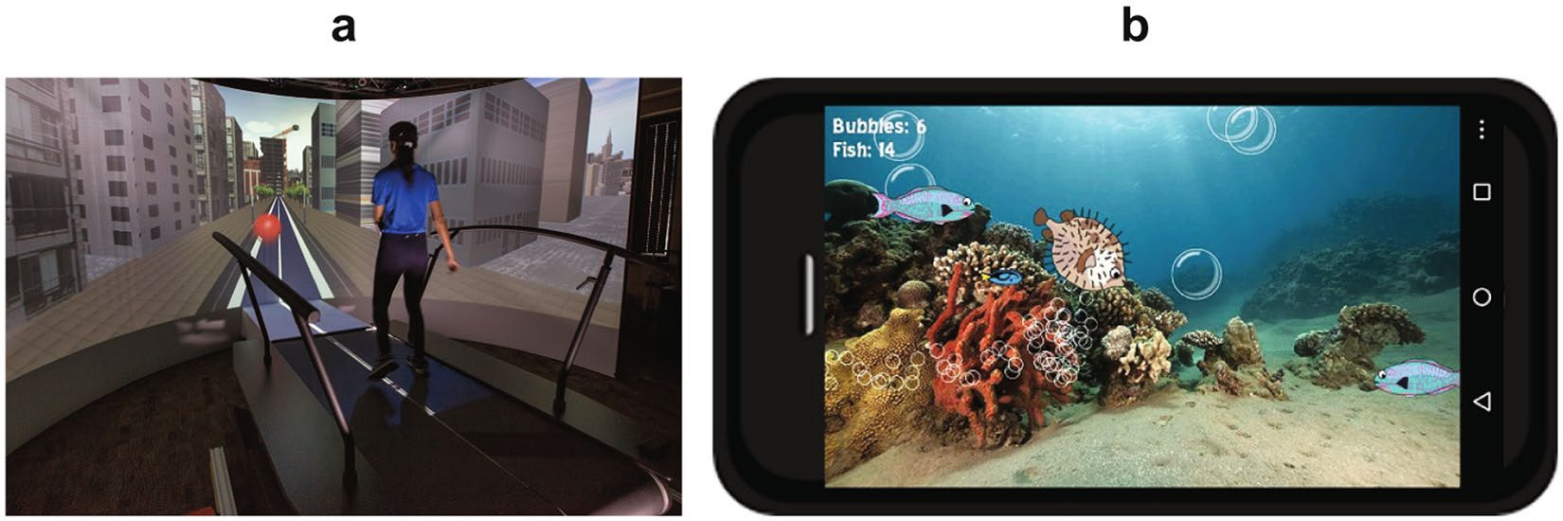
Object Negotiation and Cell Phone Tasks. (**a**) Photo of a person (not a study participant) walking in the virtual environment. The virtual environment included buildings on both sides of the road. The photo shows an approaching obstacle on the left lane and the person changing lanes to avoid the obstacle. (**b**) Schematic representation of a typical screen image participants migh have seen while performing the cell phone task. Fish and bubbles appear at random locations and times and and move horizontally (fish) orr vertically (bubbles) across the screen. When an object (fish or bubble) is tapped by the person playing the game, this object wil disappear and a score will be recorded. The scores (separately for number of bublles and fish tapped) is shown on the top left. Total Game Score was computed as the sum of these two numbers.

The walking experiments were carried out in a Motek V-Gait Virtual Reality system (Fig. 7a) that consists of a 180° semi-cylindrical visual display in front of an instrumented 1 m wide × 2 m long dual-belt treadmill (Motekforce Link, Amsterdam, Netherlands). Participant movements (kinematics) were tracked by an integrated 10-camera VICON MX motion capture system (Oxford Metrics, Inc., Oxford, UK).

Before stepping on the treadmill, 4 markers each were placed on the participant’s head, feet and pelvis, defining a 16 marker set previously established in our lab^50^. To prevent falls, participants wore a commercially available safety harness attached to an overhead support frame. This harness did not interfere with their normal movements. During all trials, the treadmill was set to run at a pre-determined comfortable speed (*v*_*w*_) for each participant (Table 1), calculated as ${v}_{w}=\sqrt{Fr\cdot g\cdot l}$, where *Fr* = 0.16 is the Froude number, *g* = 9.81 m/s^2^ is gravitational acceleration, and *l* is the leg length in meters, as we have done previously^51,52^.

There were six conditions that combined three different negotiation tasks paired with either texting or no texting. The six conditions were – No Texting-No Object Negotiation (NN), No Texting-Simple Object Negotiation (NS), No Texting-Complex Object Negotiation (NC), Texting-No Object Negotiation (TN), Texting-Simple Object Negotiation (TS), and Texting-Complex Object Negotiation (TC). Participants walked under NS, NC, TS, and TC trials for approximately 3 minutes each, whereas they walked for about 1 min during NN and TN trials, as there were no objects. Each of these six different experimental conditions were performed twice. To minimize learning effects, the order of presentation of the trials was randomized for each participant, using a counter-balanced Latin square design that balanced presentation order across all participants. Participants rested at least one minute between each trial to minimize fatigue.

Individuals performed the texting task for 20 sec while standing, after stepping on the treadmill to get acclimated to the cell phone task. During non-texting trials (NN, NS, NC), participants were instructed to hold an android touch screen smart phone (Motorola, Moto G) in landscape mode near their belly button, but the phone was turned off and participants were not required to look at the phone. During the experimental texting trials (TN, TS, TC), participants held the phone in the same position and played a standardized game (“Fish Farts”, Version 1.2) (Fig. 7b) on the cell phone that mimicked a typical texting task (game sounds were disabled). This cell phone game presented to participants randomly appearing fish and bubbles that moved across the screen. To earn points, participants had to tap them. The game tracked each user’s score in terms of the number of fish and bubbles tapped. We chose this game based on several key features. First, it is played continuously (there are no “levels” to advance through and the game never ends), thus it did not interrupt trials. Second, the game required visual attention because the fish and bubbles appeared on the screen at random locations at random time intervals. Third, the game required a “texting-like” response as it required participants to actively touch the fish and bubbles to earn points. Fourth and most importantly, Fish Farts was very easy to learn and as minimally cognitively challenging as possible (i.e., ‘don’t think – just tap the fish and bubbles’).

For the walking tasks, each participant initially practiced walking on the treadmill for 10 min, during which they were acclimated to each of the six conditions. Participants walked in a virtual environment depicting a road with two lanes (0.5 m wide each, corresponding to each treadmill belt) in a city with buildings on either side of the road (Fig. 7a). Participants were instructed to walk on one lane at a time (i.e. either left or right) and to change lanes appropriately to respond to approaching obstacles and targets. For Simple Object Negotiation (NS & TS), participants were randomly presented with obstacles (red balls) only in either lane and were instructed to always avoid these red obstacles by moving laterally to the lane that did not have the obstacle. During Complex Object Negotiation (NC & TC), participants were randomly presented with either obstacles (red balls) or targets (green balls) in either lane and were instructed to always avoid obstacles and hit targets by changing lanes as needed. Thus, Complex Object Negotiation was cognitively more challenging compared to Simple Negotiation, as individuals had to make an additional decision based on the color of the ball during Complex Negotiation.

Individuals could respond to approaching objects at any time from when they first appear to when they passed them. If a participant had not made the correct response in the time allowed, it was recorded as a failure (i.e. collision). Objects reached the center of the treadmill in two seconds after they appeared. Thus, depending on individual’s exact location on the treadmill, this gave participants approximately 2 sec to respond to each object (i.e., a little less time if they were in front of the center, or a little more if they were behind it). This ~2 sec window provided participants enough time to see the objects and alter their gait accordingly^53,54^. Feedback was provided when each object crossed the participant. Positive auditory feedback (a pleasant sound) was provided for each successful response and a combined visual-plus-auditory negative feedback (a bright flash on the screen and a loud noise resembling a collision) were provided following each failure.

### Data Collection and Processing

IQ was calculated based on the scores of the 3 sections of KBIT-2 to determine the participants skills and knowledge acquired through education and acculturation. Mean and standard deviation of IQ score was calculated across participants (Table 1).

For the PPVT test, the PEBL software calculated reaction time, defined as the amount of time taken to respond to a stimulus. Reaction times in the range of 150 and 500 msec are typically considered to be accurate responses^55^. Therefore, mean of reaction times between 150 to 500 msec was defined as Reaction Time (RT), which corresponds to a delay in the response. During the PPVT test, all participants were offered 25 stimuli, except one individual who was accidently offered seven stimuli. However, this did not affect further analysis.

For the BCST test, the PEBL software calculated several measures of executive function, including Perseverative Error, Perseverative Response, and Failure to Maintain Set. Failure to Maintain Set quantified failure to follow a rule after 5 correct responses for the same rule and thus indicated a measure of attention^56^. Perseverative Response was defined as the number of correct and incorrect response in which previous rule was followed as a percent of the total number of trials. Similarly, Perseverative Error was quantified as the number of incorrect response which would be correct for previous rule as a percent of the total number of trials. Perseverative Error and Perseverative Responses are measures of cognitive flexibility^44^, as they quantify how well participants are able to follow the previous rule.

During walking, kinematic data were recorded from markers placed on individuals. Raw kinematics data were processed using Vicon Nexus software. Additional, data processing and analyses were performed using Matlab (MathWorks, Inc., Natick, MA).

During the texting conditions (TN, TS and TC), the total number of fish and bubbles tapped and the duration of the trials were recorded for each trial. Game Score was calculated as the average number of fish and bubbles tapped per second across two trials. This was calculated by dividing the total number of fish and bubbles tapped across both trials by the total time for both trials. Game Score was used to determine the performance on the cell phone task, where higher Game Score corresponded better performance.

The task of avoiding an obstacle required participants to view and perceive the object, decide to avoid or hit it based on the color of the object, and to plan and execute the lateral shift from their current lane (i.e., current treadmill belt) to the other lane as needed. For each object encounter, Movement Time (MT) (in sec) was defined as the time taken to move laterally from the lane the participant was currently walking in to the other lane. Participant lateral movement was determined by the location of the center of their pelvis, which was calculated as the geometric centroid of the 4 markers placed on the pelvis. Movement time was calculated as the time between the appearance of the object and the shifting of the geometric centroid of the 4 pelvis markers to the other lane (i.e. across the midline of the treadmill). Responses were pooled across all objects encountered across both trials for each condition. Mean of Movement Time (Mean MT), Standard Deviation of MT (SD MT), and Percent Collision were calculated for each participant for each experimental condition involving objects (i.e. NS, NC, TS, TC). Percent Collision was defined as the total number of failures divided by the total number of (correct and incorrect) responses × 100%.

### Data Analysis

Cell phone task performance was determined on the basis of Game Score, where higher scores corresponded better performance. There were no game scores for the conditions that did not involve texting. Therefore, we compared Game Score for the three tasks that involved texting, (i.e. TN, TS and TC) using a single-factor Analysis of variance (ANOVA) with ‘Negotiation’ (No vs. simple vs. complex) as the factor. We hypothesized that Game Score would decrease with an increase in the complexity of the object negotiation task. For all statistical analyses, comparisons were considered “statistically significant” if they reached p < 0.05.

Percent Collision measured failure to avoid an obstacle. We hypothesized that Percent Collision would be higher while texting and would also increase with an increase in the complexity of the negotiation task. To test these hypotheses, we compared Percent Collision across all of the experimental conditions that involved objects (i.e. NS, NC, TS, TC), using a 2 × 2 ANOVA with Texting and Negotiation as factors.

We compared Mean MT and SD MT across conditions to quantify the effects of both the cognitive complexity of the object negotiation task and of texting. An increase in the Mean MT corresponds to a longer delay in the response, whereas an increase in the SD MT corresponds to more variable responses. We hypothesized that the Mean MT would increase due to texting and also due to an increase in the cognitive complexity of object negotiation. To test these hypotheses, we conducted two separate 2 × 2 ANOVAs for Mean MT and SD MT with Texting and Negotiation as factors.

Cognitive test statistics included mean and standard deviation of reaction times, Failure to Maintain set, Perseverative Response and Perseverative Error. Linear correlation analyses of cognitive variables with performance on cell phone (during TN, TS and TC) and negotiation tasks (during NS, NC, TS and TC) were used to quantify the extent to which the Baseline cognitive measures could predict subsequent performance on both the texting (Game Score) and object negotiation (% Collision) tasks.

## Data Availability

The datasets generated during and/or analyzed during the current study are available from the corresponding author on reasonable request.
